# Innovation in Sexuality and Relationship Education in Child Welfare: Shifting Toward a Focus on Ongoing Conversations, Connection, and Consent

**DOI:** 10.1007/s11121-022-01476-z

**Published:** 2023-01-11

**Authors:** Barbara Ball, Sharon Hoefer, Monica Faulkner, Andrea Requenes, Tia Brooks, Guadalupe Munoz, Eleni Pacheco, Cieria Poland, Carolina Salmeron, Ana Belén Zelaya

**Affiliations:** 1 Texas Institute for Child & Family Wellbeing, University of Texas at Austin, Steve Hicks School of Social Work, Austin, USA; 2 Texas Alliance of Child and Family Services, Austin, USA; 3 Healthy Futures of Texas, San Antonio, USA

**Keywords:** Child welfare, Program development, Adolescent health, Sexual health, Learning agenda in public health

## Abstract

Youth in foster care experience disproportionate rates of abusive relationships, teen pregnancy, and sexually transmitted infections (STIs). Extant research points to the need for interventions at multiple levels of the social ecology, however, there is a lack of evidence to guide the development of coordinated interventions for youth, foster parents, and child welfare professionals. The Texas Foster Youth Health Initiative (TFYHI) convened a multidisciplinary learning community to build a foundation for intervention development. The intentional learning and innovation process engaged several groups of stakeholders: young adults with lived experience (*n* = 41), foster parents (*n* = 14), and child welfare professionals (*n* = 52). Interviews, community listening sessions, and reflection exercises were designed to capture tacit and experiential knowledge and explore challenges and desired outcomes from different perspectives. Based on a thematic analysis of stakeholder perspectives, we identified overarching needs to normalize conversations about sexuality and relationships and shift away from risk-based and stigmatizing approaches. We also identified key strategies for designing coordinated interventions targeting youth, foster parents, and child welfare professionals: (1) Reflect on values about sexuality and relationships. (2) Validate youths’ need for connection. (3) Focus on strengthening youth-adult relationships and ongoing conversations. (4) Build healthy relationship skills including communication about consent, condom use, and contraception. (5) Identify teachable moments and model problem solving. (6) Use interactive approaches for sharing health information and empower youth to choose methods that fit their needs.

Youth with lived experience in foster care experience disproportionate rates of pregnancies (Dworsky & Courtney, 2010), sexually transmitted infections (STIs) (Ahrens et al., 2016), and dating violence (Herrman et al., 2016). However, efforts to address these interrelated negative outcomes have not taken root because there is little evidence to guide solutions. The Texas Foster Youth Health Initiative (TFYHI) convened a multi-disciplinary learning community that sought to develop innovative approaches to address the unique needs of youth in foster care in Texas, support foster parents and child welfare professionals, and make systemic and sustained changes in child welfare environments. TFYHI comprises partner organizations with expertise in sexual health, violence prevention, child welfare and research; initiative members include young adults with lived experience, foster parents, educators, researchers, and community stakeholders.

Texas is one of the largest child welfare systems in the country, with 45,870 children in substitute care in Financial Year 2021 (Texas Department of Family and Protective Services, 2021b). The rate of pregnant youth in foster care has risen continuously, from 6.6 pregnant youth per 1000 youth in care in 2017 to 8.3 pregnant youth per 1000 in 2021; girls in Texas foster care are five times more likely to get pregnant than their same-age peers and more than 50% of girls who emancipate from foster care become pregnant before they turn 20 (Texans Care for Children, 2022). These outcomes are mirrored in national data sets (Dworsky & Courtney, 2010).

Youth in the foster care system also experience rates of physical and sexual dating violence victimization that are three times higher than their peers (Herrman et al., 2016). Nearly one third of young women ages 16–24 years with experience in care report a history of reproductive coercion (PettyJohn et al., 2021). Compared with youth in the general population, youth with lived experience in foster care have 2–14 times greater risk of contracting STIs, including HIV (for a review see Ahrens et al., 2016).

Lesbian, gay, bisexual, transgender, and questioning (LGBTQ) youth face special barriers regarding safe exploration of sexual identity and gender expression and access to sexual and reproductive health care. LGBTQ youth are overrepresented in the child welfare system, with up to 30% of youth in foster care self-identifying as LGBTQ, compared to 11% of youth in a nationally representative sample (Baams et al., 2019). Rejection from family, strained parental relationships, and psychological and physical abuse related to their sexual identity are thought to be contributing factors to child welfare involvement. Some youth experience ongoing harassment and victimization, even after they have entered the child welfare system (Matarese et al., 2021).

## Background: Defining the Challenge

Many youth in foster care not only have traumatic histories of abuse, neglect, and loss, but also live in complex environments that impact their ability to heal from trauma, develop models for healthy relationships, connect with caregivers and peers, and access sexuality education and sexual and reproductive health care. These challenging experiences at the intrapersonal, interpersonal, and systems levels may increase their vulnerability for dating abuse, intended and unintended teen pregnancies, and negative sexual health outcomes.

Childhood trauma, including sexual trauma, is one of the strongest predictors of sexual behaviors associated with STI and HIV risk, such as early onset of sexual activity, number of sexual partners, lack of contraceptive use, unprotected sex, and transactional sex (Thompson et al., 2017). Mental health problems in adolescence, especially trauma symptoms and substance use, appear to be pathways by which childhood trauma is associated with poor sexual and reproductive health outcomes. Stigma, shame, self-blame, emotional dysregulation, and feelings of powerlessness may make it difficult for youth to negotiate conflicts, sexual consent, condom use, and birth control (Ahrens et al., 2016; Thompson et al., 2017).

Connectedness with caring adults is one of the most important protective factors in adolescents’ lives and is associated with improved sexual health outcomes and decreased physical and sexual dating violence victimization (Steiner et al., 2019). Accordingly, connectedness with caregivers irrespective of placement type has the potential to reduce sexual risk behaviors such as unprotected intercourse among youth in care (Potter & Font, 2019). Unfortunately, many youth in foster care feel disconnected from family, friends, and community, and child welfare professionals and foster caregivers report discomfort talking with youth about sexuality and relationships (Harmon-Darrow et al., 2020; Serrano et al., 2018). They also express challenges balancing adequate monitoring with giving youth opportunities to explore relationships, especially when youth have experienced significant trauma that makes them vulnerable for sexual risk behaviors and victimization (Albertson et al., 2018).

Despite the elevated risks for STIs, HIV, and unintended pregnancies, adolescent sexual development and sexual health are largely invisible in the child welfare system (Robertson, 2013). This is reflected in a lack of policies, training, and interventions, and in barriers to accessing sexual and reproductive health care. In Texas, the service plan for a child 13 years old or older should include education on healthy interpersonal relationships, healthy boundaries, pro-social communication skills, sexually transmitted diseases, and human reproduction (Texas Department of Family and Protective Services, 2021a), but there is little guidance on how to implement the service plan. To some extent, the child welfare system relegates sexual health education to the school system but sexual health education is not a required subject in Texas K-12 education. Other potential sources of sexuality and relationship education include Preparation for Adult Living (PAL) classes provided for youth 16 years and older who are likely to stay in foster care until age 18, and presentations at teen conferences hosted by the child welfare system. It is not clear whether the majority of eligible youth participate in these classes or whether the classes adequately address their needs.

In summary, existing research demonstrates a persistent and complex problem: Disproportionately high rates of teen pregnancies, abusive relationships, and STIs/HIV among youth in foster care require interventions at multiple levels. However, there is a lack of evidence from which to develop coordinated and impactful interventions for youth, foster parents, child welfare professionals, and organizations. The majority of interventions are focused on youth, yet sexual health curricula, including those marketed as curricula of choice for youth in foster care (Combs et al., 2019), typically do not discuss sexual activity from the perspective of a youth growing up in the foster care system who might be a survivor of trauma. Additionally, curricula that include fear- and shame-based content or view sexual activity as a negative choice can further stigmatize youth, trigger trauma responses, and undermine a safe learning environment (Panisch et al., 2020). Some recent efforts have shifted the focus toward capacity building for child welfare organizations, including staff training and practice and policy development (Colarossi et al., 2019; Combs & Taussig, 2021) to increase youths’ access to sexual and reproductive health information, resources, and supportive conversations with caregivers. Thus far, efforts at improving sexual health outcomes of youth in foster care have remained fragmented; there is a need to develop coordinated interventions and responsive environments that are centered on the lived experience of youth in foster care.

## Purpose of this Project

The Texas Foster Youth Health Initiative (TFYHI) adopted a framework for intentional learning and innovation (Welter et al., 2020) to create the foundation for developing coordinated interventions for youth, foster parents, child welfare professionals, and organizations. Our learning agenda was comprised of the following questions: (1) What challenges do youth in foster care experience in attaining healthy sexuality and relationships? What challenges do foster parents and child welfare professionals experience in supporting youth? (2) What are we trying to achieve? What would desired interventions and supportive environments look like? We engaged young adults with lived experience, foster parents, and child welfare professionals to gain a deeper understanding of their different perspectives and experiences. Semi-structured interviews, community listening sessions, and reflection exercises were designed to capture tacit and experiential knowledge. Based on a thematic analysis of data gathered through these learning activities, we identified assumptions that need to shift in order to support youth in reaching desired sexual health outcomes and built a framework for intervention design rooted in a systems approach.

## Methods: Designing the Listening and Learning Process

The intentional learning process was led by TFYHI staff with expertise in sexual health, violence prevention, and child welfare and include researchers, educators, and advocates. Project staff engaged stakeholders; generated learning questions; facilitated interviews, community listening sessions, and other activities; and continuously reflected on insights in monthly meetings.

### Engaging Stakeholders

Youth in foster care live in a variety of placements, ranging from relative and non-relative foster families to congregate care settings, such as emergency shelters, group homes, and residential treatment centers. Their caregivers include foster parents, staff in congregate care facilities, caseworkers, judges, health and mental health professionals, advocates, and mentors. Therefore, the intentional learning process for this project engaged young adults with lived experience, foster parents, and child welfare professionals representing a wide range of settings and roles.

#### Young Adults with Lived Experience in Foster Care

Engagement of young adults with lived experience occurred throughout the project. We first approached organizations serving youth with history in foster care and foster care liaisons in Texas colleges to recruit young adults for individual interviews. Twenty-seven young adults between the ages of 18 and 30 (*M* = 23.5, SD = 2.9) participated in semi-structured interviews lasting between 45 and 90 min. In a second, separate step we recruited young adults to participate in an ongoing consultant group with monthly online meetings lasting 60–90 min. Fourteen young adults between the ages of 18 and 25 (*M* = 20.3, SD = 2.8) participated in a consultant group with a 1-year commitment. Young adults received stipends for their participation in interviews and in the consultant group. Demographic information for young adults participating in interviews and the consultant group is provided in Table 1.

**Table 1:** Demographic characteristics of young adult participants

Characteristic	Interview participants		Consultant group	
	*n* = 27	%	*n* = 14	%
Race/ethnicity				
Non-Hispanic White	8	29.6	3	21.4
Hispanic White	8	29.6	7	50
Hispanic Multiracial	6	22.2		
African American	4	14.8	4	28.6
American Indian	1	3.7		
Gender				
Cisgender female	22	81.5	14	100
Cisgender male	4	14.8		
Transgender female	1	3.7		
Sexual Orientation				
Straight	17	63		
Gay/lesbian	4	14.8		
Bisexual	6	22.2		

Information about sexual orientation was not collected for participants in the consultant group

#### Foster Parents

We recruited foster parents to participate in an ongoing consultant group with monthly online meetings. Outreach occurred through child placing agencies, foster parent associations, and child welfare judges. Several local recruitment presentations were provided. Fourteen foster parents between the ages of 35 and 50 participated in a consultant group with a 1-year commitment. Participants were predominantly female (*n* = 12; 85.7%); 71.4% (*n* = 10) identified as white, 21.4% (*n* = 3) as African American, and 7.1% (*n* = 1) as Hispanic. Out of this group, four foster parents participated in individual interviews. Foster parents received stipends for their participation in interviews and in the consultant group.

#### Child Welfare Professionals

We reached out to child welfare professionals from a variety of positions within the child welfare system to participate in community listening sessions. Fifty-two child welfare professionals representing urban, suburban, and rural areas of Texas participated in 10 listening sessions, each lasting 60 min. Participants from public and private child welfare agencies and child welfare courts included organizational leaders, administrators, direct care staff, and mentors and were predominantly female (*n* = 41; 79%).

### Listening and Learning Activities

Listening and learning activities included semi-structured interviews with young adults and foster parents and community listening sessions with child welfare professionals. All conversations were conducted online, guided by the learning agenda questions. In addition, young adults and foster parents explored challenging situations and real-life scenarios in their monthly online consultant group meetings. Consultant group meetings were supported with Jamboard, a free and accessible online platform where participants can collaborate in real time and respond to prompts by posting notes, comments, and images anonymously. Young adult and foster parent groups met separately, but had opportunities to exchange their perspectives and ideas.

The primary authors were present in all interviews, community listening sessions, and consultant groups. Individual interviews were recorded and transcribed verbatim. In community listening sessions, two facilitators alternated between guiding the conversation and taking detailed meeting notes that aimed to capture the participants’ words in as much detail as possible. The facilitators completed and exchanged meeting notes immediately after the sessions to ensure accuracy. Meeting notes were de-identified prior to analysis. In consultant groups, participant ideas were recorded anonymously on Jamboard. All study procedures were approved at the primary author’s institution.

### Analyzing Stakeholder Perspectives

Throughout the learning process, the TFYHI project team explored how the perspectives of young adults, foster parents, and child welfare professionals aligned or differed. The two primary authors, who are both experienced in qualitative data analysis, conducted a thematic analysis and shared developing themes with the TFYHI project team in weekly meetings. In keeping with best practices in thematic analysis (Braun & Clark, 2012), the primary authors began by reviewing transcripts and notes from interviews, community listening sessions, and consultant group activities to familiarize themselves with the different perspectives of youth, foster parents, and child welfare professionals. They worked collaboratively to develop a coding system that was informed by the learning agenda and captured the data. Each data set was coded line-by-line by two researchers in dedoose and differences in coding were discussed in weekly meetings. Subsequently, data segments were interrogated and summarized with succinct statements leading to the development of themes. Themes were reviewed, first for each stakeholder group, and then across stakeholder groups to explore how their perspectives differed or aligned. TFYHI team members who had participated in or facilitated learning activities provided feedback and input for thematic analysis. During the iterative process of defining and refining themes, the team consistently linked themes with excerpts from the raw data to maintain transparency and trustworthiness. In a last step, the team integrated themes across stakeholders to develop a roadmap for coordinated intervention development.

## Findings: Stakeholder Perspectives of Challenges and Desired Outcomes

The analysis of stakeholder perspectives revealed challenges and desired changes in four areas related to attaining sexual health: Cultural and system values about sexuality, relationship models and skills, youth-adult connection, and scope of sexual health education and resources.

### Cultural and System Values about Sexuality

#### Challenge: “Sexuality Is a Subject You Do Not Touch”

Child welfare professionals shared the assessment that sexuality and relationship education for youth was needed, yet they also described confusion about responsibilities, lack of evidence-based practices, and limited resources. Caseworkers hoped that caregivers would talk with youth about sexual health and vice versa. Ultimately, they were unsure whether conversations about sexual health were happening at all and whether youth had any access to medically accurate sexual health information and resources. Child welfare professionals repeatedly described sexuality as “a subject you don’t touch.” While they agreed that both professionals and foster parents should be able to separate personal values concerning sexuality and relationships from their responsibility toward youth in their care, they recognized that many child welfare organizations and foster parents were deeply embedded in faith-based communities. They also acknowledged that caregivers who were very uncomfortable talking about sexuality and relationships might have a hard time being a resource for youth or even do harm.

The struggles among child welfare professionals and foster parents on whether and how to talk about these topics deeply affected youth. A majority of young people in consultant groups and individual interviews shared that they had had no one to talk to about sexual health and relationships. They concluded that it seemed to be a taboo topic for caregivers, possibly because of “bad experiences that people have had, it could also be just a religious thing or a belief thing.” Even if conversations did happen, adults generally gave youth the impression that sex was a bad thing and youth felt judged, rejected or accused. LGBTQ youth tended to struggle the most, as they felt their identities were suppressed or rejected. One of the young adult consultants shared a common experience:

“I grew up in a bunch of religious homes, so I heard a lot of ‘don’t do it till you’re married, it’s a sin. Let’s not talk about it,’ it was very taboo to discuss. I read a lot of romance books that weren’t necessarily age appropriate. So that’s where I got my sexual education from, not the adults around me unfortunately.” (Young adult, female)

#### Desired Outcome: “Sex Is Normal and Healthy if Precautions Are Taken”

The young adults emphasized repeatedly that what they wanted most were open and honest conversations about sexuality, relationships, and health. They wanted it to be clear that “sex is not necessarily bad; sex is normal and healthy if precautions are taken.” They wanted other youth to know that they should not be afraid to talk and ask questions, and they wanted caregivers to be supportive without making assumptions or judging youth.

Foster parents and other caregivers were clear that they needed to work through their own discomfort in order to create a non-judgmental and safe space for youth. One of the foster parents described how she works to set aside her own fears in conversations with youth.

“I start with correct names for body parts. We talk about condoms. They ask if they are allowed to buy them – I tell them yes. We talk about birth control. If a child is willing to open up and trust you enough to ask questions, you need to put your own fears aside to talk about it. My kids are very open. There is nothing they can say that will shock me! I’ve gained the skillset of having a poker face to not react.” (Foster parent, female)

In order to develop confidence for conversations about sexuality, foster parents and child welfare professionals, from administrators to direct care staff, asked for support and clear guidance, including sample language, scripts, and consistent messages. They also consistently asked for help understanding gender and sexual identities and LGBTQ inclusive language. Foster parents pointed out that they would benefit from peer support to work through discomfort and problems that might occur in these potential new conversations. They wanted to know that “it’s okay to be unsure, it’s okay to not know what you don’t know, it’s okay to be uncomfortable with a teen being sexually active” and figure out how not to let personal beliefs color the conversation.

### Relationship Models and Skills

#### Challenge: “Love = Abuse” and “Sex = Connection”

Young adults, foster parents, and child welfare professionals concurred that many youth in foster care had grown up with inconsistent or abusive parenting and lacked models for healthy relationships. However, there were subtle differences in how these stakeholders understood the impact of trauma on relationships and how they sought to cope with the challenges. Many youth grew up believing that violence and abuse were normal. “For me a lot of violence is what I considered healthy. Verbal abuse, emotional abuse seemed normal for me, when in reality that’s toxic” (Young adult, female). They also shared a common belief that “if a guy tells you he loves you, it’s time to go the whole way” (Young adult, female). The lack of connection with peers and community had youth looking for “love, even if it is in all the wrong places” (Young adult, transgender female). This was especially true during periods of intense vulnerability, such as placement instability, emancipation from care, and homelessness. In this context, sex or wanting to have a baby was a way for youth to affirm a connection. An alumni of foster care, who is now a mentor for youth in care, explained:

“I’ve noticed, even if it’s not a good relationship we tend to stay in it because that’s the person who has been there. It’s about the comfort and the consistency and the fact that they [partners] give them the love that is missing from their life. When we see a guy that says, ‘I wish you would do this,’ we think we need to have sex so he will stay.” (Young adult, female)

Foster parents recognized “the high value placed on being in a relationship because they come from this place of feeling unwanted,” but they were most concerned with what they saw as youths’ lack of boundaries and the risks of dating and sexual relationships. Caregivers were keenly aware that while they identified the lack of boundaries as a problem, youth did not. From the adults’ perspective, the urgency to be in a relationship resulted in youth making impulsive decisions; they found it difficult to accept youths’ choices when those choices appeared to undermine their placement, education, employment, housing, and financial stability. Child welfare professionals cited many situations when they were having to monitor and “regulate” risky sexual behaviors or move youth to more restrictive placements. They described their efforts to help youth understand their trauma and fears of abandonment and instill a sense of self-worth, but acknowledged limited success.

Youth in turn experienced caregivers to be controlling or even punitive, which led them to keep questions to themselves and their relationships a secret. Several young adults shared in interviews that they had been kicked out of placements for sexual behaviors, feared having a negative record, or felt that their sexuality was suppressed. They also noted that they felt ashamed asking for contraceptives or STI testing. These challenges spotlighted an environment that was reactive and emergency-based, and in which sexuality was only talked about when safety concerns were triggered.

#### Desired Outcomes: “Help Youth Learn from Mistakes”

The description of challenges demonstrated the negative impact of equating dating and sexual behaviors with risk, as this resulted in controlling or punitive responses from caregivers and child welfare organizations ultimately preventing youth from exploring and learning from mistakes. Youth overwhelmingly wanted caregivers to know that working through trauma and “getting better isn’t a straight path. We’re bound to make mistakes and we deserve support through those, too” (Young adult, female). One of the alumni who had gone on to work with young people in child welfare and juvenile justice put it this way:

“Understand that they're gonna make mistakes, but teach off of those mistakes and build from there. This happened while you were doing x, y, and z. What could have gone differently? What are the other options or choices that you had? Have them process it and then work from there.” (Young adult, female)

Foster parents and child welfare professionals emphasized that they needed training on trauma-informed strategies to help youth set healthy boundaries, regulate emotions, and build skills for healthy relationships. They recognized that they were currently focusing on risk behaviors that were really “the tail end of the problem” and wondering how they could be more proactive.

### Youth-Adult Connection

#### Challenge: “Information Without Connection Does Not Absorb”

Youth also noted that education, whether it was provided at school or through the child welfare system, was often delivered in a presentation format that left them unengaged and unable to absorb information. Without a safe space and relationships with facilitators and peers, they were not able to interact with the material and apply it to their lives. “I’ve been to a lot of presentations at [teen] conferences, they’re kinda boring. They’re usually just a person with a PowerPoint reading off the slides for an hour.” (Young adult, male). Most importantly, talking about relationships and sexuality in environments that felt unsafe could be triggering.

“School did the warning signs. They showed us this little presentation on what are signs of abuse. I knew all the signs already. I lived it. So, I think a lot of the [group home] girls felt uncomfortable with that, because it was just too much, and it was like people around us were just laughing and didn’t take it seriously.” (Young adult, female)

Caregivers acknowledged that it was hard to talk about sexuality without having a trusting, safe, and lasting relationship with the youth. In some cases, they struggled with assessing what youth did or did not know about sexual health. In addition, the fear of re-traumatizing youth by talking about sexuality contributed to hesitancy to talk about these topics.

“I think as far as the foundation you’ve got to start those hard topics early. And a lot of times we don’t have that with kids that are just coming into care. I mean a kid in care, if they don’t feel safe asking questions you’re not going to get anywhere. Because you don’t know what’s going on in their minds.” (Foster parent, female)

#### Desired Outcome: “Connection Changes a Person’s Mindset”

The discussion of challenges made it abundantly clear that youth in foster care were seeking connections. They looked for connection with caregivers and guidance on how to navigate sexual and dating relationships and pondered who they might trust with questions. A school nurse, a foster parent, a caseworker, a friend? From their perspective, conversations with caregivers went awry when caregivers responded based on their own value system, needs, and fears rather than listening to the youth. In contrast, when caregivers were attuned to the needs of the youth, ongoing conversations made an enormous difference and deepened the connection with the caregiver. Young adults emphasized that they needed love and support, especially when they struggled with peer pressure, abusive relationships, rejection, and other questions.

“I think that even sitting down and talking to a child that's hurt can start a positive relationship. So, it's kind of like even that small little conversation can change a person's mindset, or maybe help them open up a little bit more every day.” (Young adult, female)

Youth and caregivers concurred that building a connection required moving past defensiveness and slowly opening up. It also required an understanding on the part of the foster parent that youth will be independent and make their own choices, and that foster parents must therefore relinquish some control. “You want these children in your home to make the choices that you’re trying to instill, and then your kids are trying to break free and want to be independent, and yet they have things they need to know.” (Foster parent, female).

### Scope of Sexual Health Education and Resources

#### Challenge: “Education Is Limited to Warning Signs”

A majority of youth talked about missing out not only on conversations with caregivers, but also sexual health education in school. Growing up, they did not have basic information about puberty or sexual and reproductive anatomy. “I didn’t even know why I had a period. I didn’t learn any of that until late in high school,” shared one of the young adults. The lack of knowledge felt embarrassing. “When I heard the term birds and bees, I didn't know what it was. I did feel embarrassed because I didn't know and I suspected that I should know.” (Young adult, female) Furthermore, they concurred that education, when available either at school or in the child welfare setting, focused on avoiding risk and recognizing warning signs of abuse. In child welfare, sexuality was primarily seen through the lens of trauma, victimization, and perpetration.

“I didn’t know where my vagina was until I was 17. I didn’t know any of that stuff until I started exploring. We were in girl group homes. Why didn’t we talk about the female body? They don’t teach you how people have sex. And it was always more related to avoiding sexual violence than enjoying each other.” (Young adult, female)

#### Desired Outcome: “Youth Can Make Their Own Decisions”

There was consensus among young adults, foster parents, and child welfare professionals that educational programs needed to be developmentally appropriate and ongoing. They asked for resources to be tailored to the experiences of youth in care and specifically suggested that interventions focus on healthy relationships, boundaries, and safe and consensual sex in the context of real-life scenarios. Child welfare professionals also noted the need to specifically engage young men in conversations about consent, responsibility for birth control, pregnancy, and fatherhood. In addition, they emphasized that developing a sense of self-worth was important for preventing abusive relationships and helping youth find options to cope with trauma and loss that did not include sexual activity.

“I think it’s so important to have sex education constantly being discussed: different topics around safe sex, self-worth, and discussions with partners. I think self-appreciation, responsibilities, and opportunities to find self-happiness are important.” (Young adult, female)

Young adults indicated that they not only wanted comprehensive information about STI/HIV prevention and contraception, but most of all they wanted to have choices. They suggested that educational programs should be delivered by people that were relatable, had shared experience, and were not in a position of authority over the youth.

“I think it would be helpful to teach all things about sexual health, whether there are people that want to do abstinence or different kinds of birth control. And there should be information about different methods of sexual protection so the kids can make their own decision. I think trying to force a kid, especially in an environment of foster care where they have so little that they control anyway, to act a certain way when it comes to sexual health is unrealistic and not helpful.” (Young adult, male)

Given the reality that youth in care are surrounded by a host of professionals and caregivers, it was particularly important for organizational leaders to ensure that everyone, including foster parents, direct care staff, therapists, and health care providers, was on the same page, gave youth consistent messages, and worked together as a team. They suggested attending trainings together and seeking assistance from community partners to better understand the culture and diversity of youth.

## Discussion: Key Strategies in Intervention Design

**Fig. 1:**
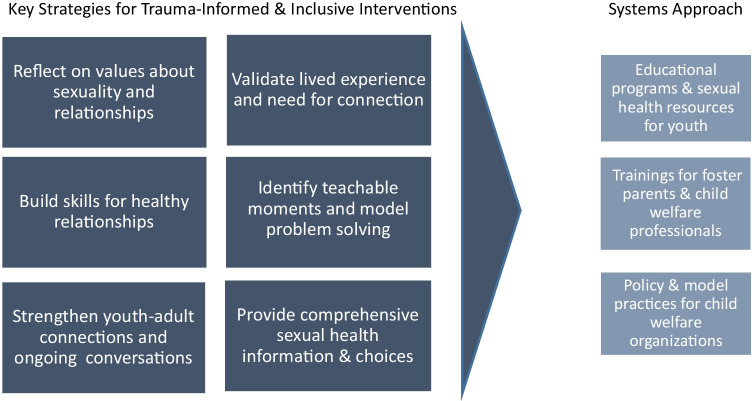
Framework for coordinated, trauma-informed, and inclusive sexual health interventions in child welfare

The Texas Foster Youth Health Initiative (TFYHI) embarked on an intentional learning and innovation process (Welter et al., 2020) to develop a framework for intervention design. Listening to stakeholder perspectives confirmed the need for trauma-informed and inclusive interventions at multiple levels of the social ecology, including youth, foster parents, child welfare professionals, and organizations. Youth need multiple access points for sexuality and relationship education and ongoing supportive conversations with caring adults. Organizational practices and policies in child welfare organizations need to normalize conversations about sexuality and sexual health, provide training and guidance to affiliated foster parents and professionals, and increase youth access to education and resources. Our findings extend recent studies that have begun to focus on a holistic and systems approach to the sexual and reproductive health needs of youth in the child welfare system (Aparicio et al., 2021; Colarossi et al., 2019). By comparing and contrasting stakeholder perspectives, we were able to identify key strategies that need to be integrated in interventions for all target populations in order to provide a consistent, coordinated, and impactful approach and achieve the desired outcomes. The following section discusses findings and outlines a framework for intervention design that is visually represented in Fig. 1.

### Reflect on Values and Create Safe and Inclusive Spaces

Our conversations with stakeholders showed that sexuality is indeed taboo and invisible in the child welfare system (Robertson, 2013) and primarily viewed through the lens of risk and victimization or perpetration. This view of sexuality creates a ripple effect that leads to a lack of consistent sexuality and relationship education; discomfort on the part of foster parents and caregivers when talking about sexuality; and reactive, controlling responses to youth sexual behaviors. Instead, a trauma-informed view of sexuality would acknowledge past experiences of abuse and highlight the potential for healing, and safe and consensual sexual experiences and relationships (Panisch et al., 2020). Therefore, innovative interventions need to provide opportunities for child welfare professionals and caregivers to reflect on systemic, cultural, and personal values that restrict their understanding of sexuality and youths’ developmental needs for exploration, self-expression, and learning. Values reflection can also increase caregivers’ empathy with the diverse identities of youth and their sexual health needs.

### Validate the Need for Connection

The search for connection was identified by youth and adults as a driving force that sometimes led youth to rush into intimate relationships, want to get pregnant and start a family, and hold on to partners. Previous studies have demonstrated that about 35% of young women with history in foster care who became pregnant at age 19 had wanted to become pregnant (Dworsky & Courtney, 2010). From the perspective of child welfare professionals and caregivers, these behaviors and decisions are often considered risky, maladaptive, and impulsive. Where youth seek connection and love, adults emphasize boundaries and responsibilities. These divergent perspectives, if not given consideration, can constitute a hurdle for empathic conversations and effective interventions and deepen distrust among youth and their caregivers. Youths’ lived experience and need for connection should be validated and centered in intervention design. Opportunities for connection can be integrated into multi-session interventions for youth through small group activities, which provide a space for youth voice, conversation with peers, and sharing of different perspectives, in contrast to the prevailing didactic delivery of information.

### Build Skills for Healthy Relationships

Stakeholders confirmed the high prevalence of unhealthy and abusive relationships in the youths’ lives (Herrman et al., 2016) and the lack of positive relationship models. Youth in foster care need to see and feel examples for healthy relationships. As such, caregivers have an important role in demonstrating clear communication, boundary setting, and self-advocacy in daily interactions. Therefore, interventions for youth and adults alike need to provide opportunities to model and practice communication and relationship skills in the context of real-life scenarios. Youth specifically need opportunities to practice talking about consent, safe sex, and contraceptive choices with a partner.

### Identify Teachable Moments and Model Problem Solving

Learning from experience and mistakes is important for all youth, yet the child welfare system in its efforts to protect youth and avoid risks is not well positioned for allowing youth to explore relationships and sexuality (Pokempner et al., 2015). Our work showed that “problem behaviors” can quickly result in placement disruptions, more restrictive levels of care, and youth feeling stigmatized. Youth want to know that they deserve healthy and safe relationships and that they can work through difficult situations and make different choices in the future. They ask caregivers to help them problem solve and build skills for healthy relationships and safer, consensual sex. Trainings for caregivers and child welfare professionals can reframe difficult situations as learning opportunities and model trauma-informed strategies for setting boundaries and supporting youth.

### Focus on Ongoing Conversations

Young adults with lived experience strongly emphasized the importance of ongoing conversations about sexual health. They also articulated that information provided outside of trusting relationships was difficult to absorb and process. Therefore, building strong youth-adult connections should be a cornerstone of sexual health interventions (Dworsky & Courtney, 2010). Child welfare professionals and caregivers need guidance and skill-building trainings (Albertson et al., 2018; Serrano et al., 2018) to increase their confidence for trauma-informed sexual health conversations. Building trust to share sensitive personal information and questions takes time, but youth want and value honest and trustworthy information (Aparicio et al., 2021). Our group of young adult consultants participated in developing a training for caregivers and scripted sample conversations to demonstrate strategies for opening up a conversation that felt affirming and validating to youth.

### Provide Comprehensive Information and Choice

Many youth perceived foster care as an environment that afforded them little control over their lives and limited their choices and normal age-appropriate activities. Not having a sense of control creates adversarial relationships with caregivers and undermines learning how to make important decisions about personal relationships and health. Therefore, sexual and reproductive health information needs to be provided in a format that allows youth to make choices that fit best with their current needs. It is critically important that youth in foster care understand their rights, have medically accurate information, and feel empowered in making choices for their sexual and reproductive health care needs.

## Limitations

Eliciting stakeholder perspectives generated evidence from which to develop coordinated and impactful interventions for youth, caregivers, child welfare professionals and organizations. However, this paper has several limitations. Our findings are specific to youth, caregivers, and organizations in Texas communities, and cannot be generalized. Furthermore, stakeholders who participated in interviews, community listening sessions, and consultant groups were convenience samples and did not represent the populations of youth in foster care, foster parents, or child welfare professionals. Additional research is needed to specifically explore the sexual and reproductive health needs of young men and LGBTQ youth who were underrepresented in our learning process.

## Conclusion: Reflections on the Intentional Learning Process

The intentional learning process afforded us many insights about challenges, desired outcomes, and key strategies for improving optimal health outcomes that extend existing research in child welfare. Most importantly, the process forged a vibrant learning community. Hearing directly from stakeholders helped TFYHI partners understand the lived experience of youth and the child welfare environment in new and immediate ways and appreciate their strengths and search for solutions. Engaging stakeholders in the listening and learning process opened doors for ongoing collaborative work in intervention design and testing, and a sense of ownership and commitment. Stakeholders, including young adults with lived experience, foster caregivers, child welfare professionals, and organizations, continue to work with TFYHI project staff in the next phase dedicated to designing and testing interventions.
